# Difficult Labor in Twins

**Published:** 1879-03

**Authors:** J. Lovick Sappington

**Affiliations:** West Point, Ga.


### DIFFICULT LABOR IN A CASE OF TWINS.

By J. LOVICK SAPPINGTCN, M.D., West Point, Ga.

Called December 11th, 1878, to see a negress primapara; arrived at half-past 11 o'clock; found that she had been in active labor seventy-five hours; condition good for the length of her suffering; lower extremities and body of a child external to the vulva, and had been for twenty hours, and another in utero.

Bringing down the arms and exerting gentle traction the right shoulder came under the pubis; a few strong pains and traction having no effect, a more careful examination revealed the occipital bone of the head of the first child, resting on the brim of the pelvis right side, and the same region of the head of the second child under the chin of the first, and os frontis infringing upon left lateral brim of the pelvis. Not being certain of my diagnosis, I sent for my father, Dr. J. S. Sappington, who confirmed my opinion, and assisted me out of the scrape in the following manner: Introduced my left-hand dorsum to the chest of the child, and with the points of my fingers against the vertex of the presenting head, raised it upwards, and to the mother's left, and, at the same time, bimanual pressure by my father, caused the head of the first child to come down into the pelvic cavity, and a pain coming on the child with my hand was expelled, and the next pain the second child and the placenta came away; both children still-born, the mother making a fine recovery.

In my reading I recollect but one similar case (recorded in Ramsbotham's Obstetrics, by Keating, page 468,) and in that the first was decapitated, and the second delivered with the forceps. I am frank to confess that I do not like to cut.

